# Attitude and help-seeking behavior of the community towards mental health problems

**DOI:** 10.1371/journal.pone.0242160

**Published:** 2020-11-12

**Authors:** Yonas Tesfaye, Liyew Agenagnew, Gudina Terefe Tucho, Susan Anand, Zewdie Birhanu, Gutema Ahmed, Masrie Getenet, Kiddus Yitbarek

**Affiliations:** 1 Department of Psychiatry, Jimma University, Jimma, Oromia, Ethiopia; 2 Department of Environmental health sciences and Technology, Jimma University, Jimma, Oromia, Ethiopia; 3 School of Nursing and Midwifery, Jimma University, Jimma, Oromia, Ethiopia; 4 Department of Health, Behavior, and Society, Jimma University, Jimma, Oromia, Ethiopia; 5 Department of Biostatistics and Epidemiology, Jimma University, Jimma, Oromia, Ethiopia; 6 Department of health policy and management, Jimma University, Jimma, Oromia, Ethiopia; Universita degli Studi di Milano-Bicocca, ITALY

## Abstract

**Background:**

Community attitude towards mental health problems and help-seeking behavior plays a major role in designing effective community based mental health interventions. This study aimed to assess the attitude, help-seeking behavior, and associated factors of the Jimma zone community towards mental health and mental health problems.

**Methods:**

A community-based cross-sectional study design was employed. A respondent from each of the 423 systematically selected households was interviewed using a pretested, structured, and interviewer-administered questionnaire. Accordingly, a community’s attitude towards mental health problems was measured by the adapted version of the “Community Attitude towards Mentally Ill questionnaire (CAMI)” and help-seeking behavior was measured by a general help-seeking questionnaire. Data were entered into Epi-data version 3.1 and exported to SPSS version 23.0 for analysis. Bivariate and multivariate logistic regression analysis was done to determine the independent predictors of the outcome variable.

**Results:**

Among the total 420 study participants (197,46.9%) of them had an overall unfavorable attitude towards mental illness. The majority (153,36.4%) of the study participants agreed on avoidance of anyone who has mental health problems and (150,35.7%) participants described marrying a person with a mental health problem or recovered from the problem is foolishness. Moreover, regression analysis showed family monthly income (AOR = 0.24, 95%CI:0.06–0.91) and occupational status (AOR = 0.57, 95%CI:0.34–0.96) were found to be the predictors of community attitude towards mental health problems. The study finding also revealed a significant number of the respondents preferred non- medical treatment approaches.

**Conclusion:**

Almost half of the respondents had an unfavorable attitude towards mental health problems and the traditional and religious help-seeking intention was high. This suggests the need for designing effective community based mental health interventions to improve the general public attitude and help-seeking behavior towards mental health problems.

## Introduction

Mental health is paramount to personal well-being, family relationships, and successful contributions to society. It is pivotal to the development of communities and countries [1, 2].

Beliefs about causation and experience influence patients’ beliefs about effective treatment and determine the type of treatment sought. In Africa, mentally ill patients are often blamed for bringing on their illness, in contrast, others may see mentally ill people as victims of unfortunate fate, religious and moral transgression, or even witchcraft [3–6]. This may lead to denial by both sufferers and their families, with subsequent delays in seeking professional treatment [7].

Myths and beliefs about mental health and illness are ubiquitous in every community which influences people’s attitudes [8]. Most of the African society’s attitude towards mental illness is far from the scientific view which may negatively affect treatment-seeking and treatment adherence [9].

Of all the health problems, mental illnesses are poorly understood by the general public. Societal prejudice and bias towards mental illness threaten the provision of high-quality holistic patient care including rehabilitation [10, 11]. Moreover, the negative attitudes prevalent in the community deter mentally ill people from treatment-seeking and treatment adherence [12, 13].

People often seek medical help after they have tried all options and after symptoms have become worse and this in turn negatively affects the prognoses of treatment. Hence, assessing the community’s attitude and help-seeking behavior is essential to have an appropriate health promotion plan and scale up the publics’ utilization of mental health services, particularly in developing countries with multiethnic and multicultural society [9, 14, 15].

No group of human beings can claim to be immune from developing a mental illness and it poses a serious challenge to global community health and development. However, mental health issues have been little investigated in less developed regions of the world and limited information is available about the perception and the attitude of the public regarding mental health problems in the emerging nations [16].

In Ethiopia, only a few studies are available on help-seeking behavior. Available studies reported that only 7% of persons with severe mental disorders living in rural communities sought help from mental health professionals. Likewise, another community-based study reported traditional healers preferred over modern sources of support for mental illness [17–19].

To the best of our knowledge, there is a lack of studies on the rural community attitude towards mental health problems and help-seeking behavior in developing countries, this figure is much worsened in the context of Ethiopia. Evidence from previous studies has shown that the community’s attitude and help-seeking intention towards mental health problems were malicious and preferred non-medical treatment approaches. However, these studies were mainly institution-based, the respondents were mostly students, health professionals, and urban residents’ focus [10–12, 20]. This evidence highlights the importance of researching rural public attitudes and help-seeking intention toward mental health problems. The findings will also help health planners and policymakers design evidence-based useful community mental health interventions. Hence, the present study aimed to assess the public’s attitude and help-seeking behavior toward mental health problems.

## Materials and methods

### Study setting and period

The study was conducted in Jimma zone, Seka Chekorsa district/woreda. Jimma zone is divided into 20 districts and one town administration with 545 Ganda (lowest administrative unit in Oromia region); among these, 515 are rural. The total population of the zone was 3,209,127 in 2017 [21]. In the zone, there are four primary hospitals, 20 health centers, and 117 health posts. Additionally, there are 36 private and 3 NGO clinics, 65 private rural drug vendors [22]. One thousand twenty-four health extension workers are serving the population of this area. Seka Chekorsa district is located at 20km from Jimma town, and the community has 30 Gendas with a total population of 208,096 [23]. There was one primary hospital, nine health centers, and 35 health posts found in the district. Seka Chekorsa population predominantly belonged to the Oromo ethnic group and Islam religion follower. The study was conducted from March 1 to 22, 2020.

### Sample size estimation

Sample size was calculated using single population proportion formula, considering the estimated proportion of attitude and help-seeking behaviour of 50% to get the maximum sample size, 95% confidence level, 5% margin of error, n = (zα /2)^2^ P(1-p)/ d^2^, hence n = (1.96)^2^ x 0.5 (1–0.5) / (0.05)^2^ = 384. finally, with the addition of a 10% contingency for non-response, the final sample size was 423 households.

### Population and sampling procedures

A community-based cross-sectional survey was conducted. Initially, Seka Chekorsa district was selected from the 20 districts in the zone through a simple random sampling lottery method. Out of the 30 Gandas in this district, nine were selected by lottery method based on the WHO guideline for sample size estimation [21]. The number of sampled respondents from each Ganda was determined using proportional allocation to the total number of households in each selected Ganda. A systematic random sampling technique was used to select the study units. The periodic interval (K) was calculated using the formula K = N/n, whereby N is the total households in the selected Ganda (1555). N is the estimated sample size (423). Accordingly, every four households were included in the study. The first study unit was selected by a lottery method between the 1^st^ and 4^th^ households. Finally, randomly selected household members age 18 and above living in the district for six months or more in the selected households were responded to the interview.

### Data collection tools and procedures

The data collection was conducted using a structured questionnaire obtained from the standard tools and adapted to our contexts. The questionnaire consisted of sociodemographic, attitude, and general help-seeking behavior of the respondents towards mental health problems questions. Community attitude towards mental health and illness was measured by the adapted version of Community Attitude Towards Mentally Ill Questionnaire (CAMI), which is a well-validated and reliable tool with Cronbach’s alpha score of 0.87 [20, 24–26], and in this study, the score was 0.75. The respondents were asked to rate each attitude related questions on a five-point Likert scale ranging from 1 (strongly disagree), 2 (disagree), 3 (Neutral), 4(Agree) to 5 (strongly agree). Help-seeking behavior was assessed through the General Help-seeking Behavior questionnaire(GHSQ) [27], which is a validated and reliable tool with Cronbach’s alpha score of 0.85 [28], in this study the tool had a 0.78 Cronbach alpha score. The tool has been used in many research to assess the help-seeking intention of the community [27–30]. GHSQ has a seven-point Likert scale ranging from extremely unlikely to extremely likely responses. The mean score was calculated for attitude and help-seeking behavior. A cutoff of point below and above the mean score was taken to calculate the community’s percentage with favorable, unfavorable attitude and good and poor help-seeking intention [31]. Moreover, the higher scores indicated a favorable attitude and good help-seeking intention.

Trained data collectors collected the data. The data collectors’ selection was made based on their prior mental health data collection experience and proficiency in the local languages. The questionnaires were pre-tested on 5% of the population in the Mana district to ensure its clarity and consistency. The data were obtained through face to face interviewer-administered and structured questionnaire prepared in English and translated into local languages (Afaan Oromo and Amharic) and translated back to English to ensure consistency by blinded language experts.

### Data management and statistical analysis

The collected data was cleaned, coded, entered into Epi Data version 3.1, and exported to SPSS version 23 for analysis. Descriptive statistics were done to summarize the dependent and independent variables. The logistic regression analysis model was used to identify independent predictors of the outcome variable; first Bivariate logistic regression was done, and variables with p-value < 0.25 were selected as candidate variables for multivariate logistic regression analysis. Multicollinearity and Lemeshow-Hosmer test of model fitness test was done before the final model, then Variables with P value < 0.05 and 95% confidence interval odds ratio on the final model was considered as independent predictors of the outcome variable.

### Ethical consideration

The study was reviewed and approved by the Institutional Review Board (IRB) of Jimma University (IHRPGD/584/2019). An official letter of support was obtained from the Oromia Health Bureau and then from the Jimma zone health bureau. A subsequent support letter was sought from Seka Chekorsa District health office before the commencement of data collection. Respondents were briefed on the study objectives and were assured of the anonymity of their participation. The study participation was wholly voluntary and written informed consent was obtained from each respondent.

## Results

### Socio-demographic characteristics

A total of 420 study participants completed the interview successfully, giving a response rate of 99.3%. The participants’ mean age was (37.2 years, SD ± 11.9), ranging from 18 to 80 years. The majority of the study respondents were female (230,54.8%), married (345,82.1%), from rural residence (253,60.2%), Oromo ethnic group (395,94%), and Muslim (384,91.4%). The mean family monthly income was 1562.5 Ethiopian Birr (ETB) (SD ± 2769.8) (Approximately $48.00 US), and most respondents were unable to read and write (168,40%) (Table 1).

**Table 1:** Sociodemographic characteristics of respondents at Jimma Zone, Seka Chekorsa district Oromia, Ethiopia, March 2020.

Variables	Characteristics	Frequency	Percentage
Sex	Male	190	45.2
	Female	230	54.8
Residence	Urban	167	39.8
	Rural	253	60.2
Birth order	First	183	43.6
	Second	102	24.3
	Third	60	14.3
	4^th^ or more	75	17.9
Ethnicity	Oromo	395	94.0
	Amhara	12	2.9
	Others*	13	3.1
Religion	Muslim	384	91.4
	Orthodox	24	5.7
	Others^	12	2.8
Educational status	Unable to read and write	168	40.0
	Read and write	81	19.3
	Primary school (1–8)	112	26.7
	Secondary school (9–12)	49	11.7
	Diploma	6	1.4
	Degree and above	4	1.0
Marital status	Single	30	7.1
	Married	345	82.1
	Divorced	12	2.9
	Widowed	33	7.9
Occupational status	Farmer	219	52.1
	Merchant	61	14.5
	Daily laborer	18	4.3
	Housewife	100	23.8
	Employed	22	5.3

*Other ethnicities include Kefa, Yem, Dawro, Gurage, Silte

^Others religion includes Protestant, Catholic, Wakefeta

### Attitude towards mental health and mental health problems

Most of the study participants agreed with the best way to handle the mentally ill patients is to keep them behind locked doors (154,36.7%), likewise (190,45.2%) were neutral to their response on mental illness is not an illness like any other illness. The majority (125,29.8%) respondents opined mentally ill patients are a burden on society, and (53,36.4%) responded it is best to avoid anyone who has mental problems. Similarly, (158,37.6%) described it as shameful to mention someone in the family who has a mental illness. Moreover, (150,35.7%) participants replied a woman or a man would be foolish to marry someone who has suffered mental illness even though he/she seems fully recovered. Furthermore, (167,39.8%) agreed that anyone with a history of mental problems should be excluded from taking public office, and (181,43.1%) agreed the mentally ill should be denied their rights, but (148,35.2%) were neutral to all mentally ill patients are dangerous (Table 2).

**Table 2:** Attitude of the respondents towards mental health and mental health problems at Jimma Zone, Seka Chekorsa district Oromia, Ethiopia, March 2020.

Characteristics	Frequency (percentage)					
	Strongly Disagree	Disagree	Neutral	Agree	Strongly Agree	Mean (SD)
One of the leading causes of mental illness is a lack of self-discipline and will power	78(18.6)	92(21.9)	103(24.5)	98(23.3)	49(11.7)	2.88(1.28)
The best way to handle the mentally ill is to keep them behind locked doors	31(7.4)	41(9.8)	56(13.3)	154(36.7)	138(32.9)	3.78(1.21)
A person showing signs of mental disturbance, shouldn’t get treatment in a hospital	21(5.0)	34(8.1)	43(10.2)	170(40.5)	152(36.2)	3.95(1.11)
Mentally ill patients need the same kind of control as a young child	132(31.4)	99(23.6)	68(16.2)	81(19.3)	40(9.5)	2.52(1.35)
Mental illness is not an illness like any other	17(4.0)	36(8.6)	49(11.7)	190(45.2)	128(30.5)	3.90(1.05)
The mentally ill should be treated as outcasts of society	33(7.9)	61(14.5)	63(15.0)	187(44.5)	76(18.1)	3.50(1.17)
Emphasis should be placed on protecting the public from the mentally ill	78(18.6)	155(36.9)	105(25.0)	51(12.1)	31(7.4)	2.53(1.14)
Mental hospitals are an outdated means of treating the mentally ill	159(37.9)	124(29.5)	84(20.0)	33(7.9)	20(4.8)	2.12(1.14)
Mental health problems last forever	29(6.9)	52(12.4)	144(34.3)	116(27.6)	79(18.8)	3.39(1.13)
The mentally ill do deserve our sympathy	27(6.4)	63(15.0)	70(16.7)	131(31.2)	129(30.7)	365(1.23)
The mentally ill are a burden on society	56(13.3)	92(21.9)	63(15.0)	84(20.0)	125(29.8)	3.31(1.43)
It is best to avoid anyone who has mental problems	39(9.3)	44(10.5)	80(19.0)	153(36.4)	104(24.8)	3.57(1.22)
The mentally ill shouldn’t be given responsibility	99(23.6)	113(26.9)	81(19.3)	90(21.4)	37(8.8)	2.65(1.28)
The mentally ill should be isolated from the rest of the community	57(13.6)	69(16.4)	72(17.1)	152(36.2)	70(16.7)	3.26(1.29)
It is shameful to mention someone in your family who has a mental illness	38(9.0)	37(8.8)	81(19.3)	158(37.6)	106(25.2)	3.61(1.21)
A woman/man would be foolish to marry a man/woman who had a mental illness, even though he/she seems fully recovered	48(11.4)	76(18.1)	76(18.1)	150(35.7)	70(16.7)	3.28(1.26)
It would be a problem to live next door to someone who has been mentally ill	60(14.3)	92(21.9)	136(32.4)	99(23.6)	33(7.9)	2.89(1.15)
Anyone with a history of mental problems should be excluded from taking public office	40(9.5)	74(17.6)	75(17.9)	167(39.8)	64(15.2)	3.34(1.20)
The mentally ill person should be denied their rights	37(8.8)	60(14.3)	50(11.9)	181(43.1)	92(21.9)	3.55(1.22)
All mentally ill patients are dangerous	35(8.3)	91(21.7)	148(35.2)	98(23.3)	48(11.4)	3.08(1.11)

### Overall attitude level of the study participants

**Fig 1:**
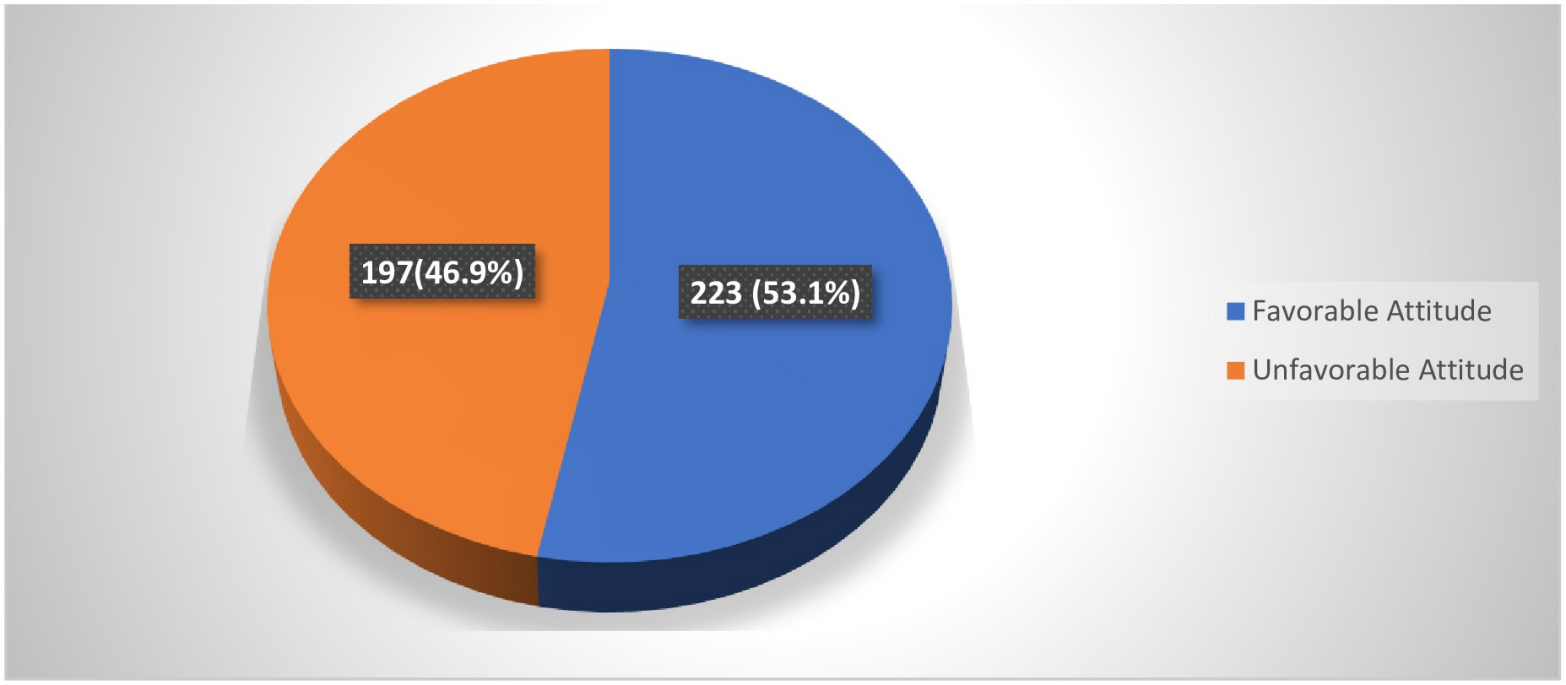
Attitude of community towards mental health problems.

The mean overall attitude score was found to be (64.8, SD± 8.7) (possible values = 20–100) with the minimum and maximum value of 28 and 89 out of 20 attitude Likert scale items. Calculating the study population’s percentage with a cutoff score below and above mean category, the overall attitude score showed (223,53.1%) of the respondents had favorable attitude and the remaining (197,46.9%) had an unfavorable attitude towards mental health problems (Fig 1).

### Help-seeking behavior of the study participants

In the event of experiencing a personal or emotional problem the majority of the respondents (145,34.5%) were most likely to seek help or advice from their partner (e.g., significant boyfriend or girlfriend, besides, one quarter (106,25.2%) respondents were likely to seek help from mental health professionals (Table 3).

**Table 3:** Help-seeking behavior of the respondents towards mental health and mental health problems at Jimma Zone, Seka Chekorsa district, Oromia, Ethiopia, March 2020.

Characteristics	Frequency (percentage)						
	Extremely unlikely	Very unlikely		neutral	likely	Very likely	Extremely likely
Partner (e.g., significant boyfriend or girlfriend)	28(6.7)	51(12.1)	34(8.1)	27(6.4)	77(18.3)	58(13.8)	145(34.5)
Friend (not related to you)	47(11.2)	55(13.1)	56(13.3)	62(14.8)	64(15.8)	98(23.3)	38(9.0)
Parent	37(8.8)	31(7.4)	18(4.3)	46(11.0)	67(16.0)	84(20.0)	137(32.6)
Another relative/family member	48(11.4)	39(9.3)	44(10.5)	131(31.2)	66(15.7)	70(16.7)	22(5.2)
Mental health professionals	51(12.1)	49(11.7)	32(7.6)	56(13.3)	106(25.2)	64(15.2)	62(14.8)
Teacher	66(15.7)	51(12.1)	111(67)	67(16.0)	65(15.5)	42(10.0)	18(4.3)
Religious leaders	47(11.2)	57(13.6)	40(9.5)	39(9.3)	110(26.2)	79(18.8)	48(11.4)
I would not seek help from anyone	184(43.8)	63(15.0)	83(19.8)	29(6.9)	18(4.3)	17(4.0)	26(6.2)

The mean overall help-seeking behavior score was found to be (4.2, SD± 1.2) with the minimum and maximum value of 1 and 6.88 respectively out of 9 help-seeking behavior Likert scale items. Calculating the study population’s percentage with a cutoff score below and above mean category, the overall help-seeking behavior score showed (163,38.8%) of the respondents had good help-seeking behavior and the remaining (257,61.2%) had poor help-seeking behavior towards mental health problems.

### Preference for help-seeking behavior

Medical treatment was not the solely preferred treatment for mental illness although it was selected as a treatment option by the majority (337,80.2%) of the study participants. Most (358,85.2%), and (256,61.0%) respondents replied medication and professional counseling can be an effective treatment for people with mental illnesses respectively, in contrary to this almost half of the participants opined that mental illness should be managed by witchdoctors and cured by marriage could be as well (Table 4).

**Table 4:** Treatment preference of the respondents towards mental health and mental health problems at Jimma Zone, Seka Chekorsa district, Oromia, Ethiopia, March 2020.

Variables	Characteristics		Frequency	Percentage
Mental illness can be treated*	Traditional	Yes	205	48.8
		No	215	51.2
	Religious	Yes	272	64.8
		No	148	35.2
	Medical	Yes	337	80.2
		No	83	19.8
Professional advice or counseling can be an effective treatment for people with mental illnesses		Yes	256	61.0
		No	164	39.0
Medication can be an effective treatment for people with mental illnesses		Yes	358	85.2
		No	62	14.8
Mental illness requires treatment from the psychiatric hospital		Yes	378	90
		No	42	10
Mental illness can be successfully managed at home by families		Yes	209	49.8
		No	211	50.8
Witchdoctors should manage mental illness		Yes	215	51.2
		No	205	48.8
Mental illness can be cured by marriage		Yes	207	49.3
		No	213	50.7

*Multiple responses were possible

Only (67,16.0%) of the respondents had sought help from a health professional for their mental health problems, among them (32,47.8%) had visited the hospital and were attended by nurses (29,43.3%). Of those who have got the services, most (35,52.2%) described the experience as extremely helpful (Table 5).

**Table 5:** Help-seeking experience of the respondents towards mental health and mental health problems at Jimma Zone, Seka Chekorsa district Oromia, Ethiopia, March 2020.

Variables	Characteristics	Response	Frequency	Percentage
Have you ever seen a health professional get help for personal/mental health problems?		Yes	67	16.0
		No	353	84.0
If yes from where?	Health center		14	20.9
	Health post		17	25.4
	Hospital		32	47.8
	Private clinics		4	6.0
Do you know which type of health professional(s) you’ve seen	Psychiatrist		4	6.0
	Psychiatry nurse		1	1.5
	Nurse		29	43.3
	Doctor		26	38.8
	I don’t know		7	10.4
How helpful was the visit to the health professional?	Extremely helpful		35	52.2
	Helpful		13	19.4
	Neutral		14	20.9
	Unhelpful		2	3.0
	Extremely unhelpful		3	4.5

### Predictors of attitude toward mental health & mental problems

Socio-demographic factors such as sex, age residence, birth order, family monthly income, educational status, marital status, occupational status, and knowledge status of the respondents were entered in the bivariate logistic regression analysis. The result has showed residence, educational level, family monthly income, and occupational status were found to have an association at P-value <0.25. After checking multicollinearity and model fitness, multivariate regression analysis was done to determine the respondent’s attitude status’s independent predictors.

Hence, those participants with family income less than 1000 ETB, were 74% less likely to have a favorable attitude than those exceeding 5000 ETB (AOR = 0.24, 95% CI:0.06–0.91). Farmers were 43% less likely to have a favorable attitude than those employed in organizations (AOR = 0.57, 95%CI:0.34–0.96) (Table 6).

**Table 6:** Predictors of mental health and mental health problems attitude of the respondents at Jimma Zone, Seka Chekorsa district Oromia, Ethiopia, March 2020.

Variables	Category	Attitude toward mental health & mental problems		COR (95% CI)	AOR (95% CI)
		Unfavorable	Favorable		
		Frequency (%)	Frequency (%)		
Family monthly Income	<1000	106(55.2)	86(44.8)	0.17(0.04–0.62) *	0.24(0.06–0.91) **
	1001–2999	81(43.5)	105(56.5)	0.27(0.07–0.99) *	0.35(0.09–1.33)
	3000–4999	7(28.0)	18(72.0)	0.55(0.12–2.52)	0.60(0.12–2.89)
	>5000	3(17.6)	14(82.4)	1	
Occupational status	Farmer	124(56.6)	95(43.4)	0.51(0.31–0.82) *	0.57(0.34–0.96) **
	Merchant	23(37.7)	38(62.3)	1.10(0.57–2.11)	1.05(0.53–2.07)
	Housewife	10(25.0)	30(75.0)	2.00(0.88–4.54)	1.56(0.66–3.67)
	Employed	40(40.0)	60(60.0)	1	1

Note:

* show P- value < 0.25,

** show variables significant at P value <0.05

^1^-Reference

## Discussion

In this study, we have evaluated the community’s attitude towards mental health problems and some associated factors.

This study has shown more than sixty percent of the respondents agreed it is best to avoid anyone who has mental health problems. Similar findings were reported from the studies done in Nigeria [32–34], India [12, 35], Iraq [36], and Saudi Arabia [37]. Indicating the ignorance and discrimination associated with mental illness in most societies, attitudes are not substantially different across the globe. Additionally, the negative public perception of patients with mental health problems is dangerous, irresponsible, and foolish might contribute to discrimination.

This study finding revealed more than half of the participants agreed a woman or a man would be foolish to marry someone who had a mental illness, even though he/she seems fully recovered. A similar belief was reflected from the studies done in Iraqi [36], Nigeria [32–34], and West Bengal India [35]. This findings validate the negative attitudes and stigma linked to mental illness are barriers to marital life.

In this study, nearly two-thirds of the participants agreed that mentally ill patients should be denied their rights. In contrary to this finding, the studies were done in Iraq [36] and India [38] showed the vast majority of the respondents agreed individuals with mental illness should have the same rights as anyone else. This could be explained by despite the policies and legislations on mental health and human rights from WHO and the UN, people still consider the mentally ill patients as cognitively impaired and aggressive, this reaffirms the need for creating awareness on mental illness.

Furthermore, in the current study, more than one-third of the respondents concurred all mentally ill patients are dangerous, which is corroborated by studies reported from Nigeria [32], Iraq [36], Saudi Arabia [37], and the rural population in India [38]. Media has an impact on the thinking, behavior, and emotions of the general population. Media’s inaccurate and exaggerated portrayal of mentally ill people as dangerous and criminals could have a negative impact on people’s attitudes towards mental health problems.

In the current study, 85.2% and 61.0% of respondents described medication and professional counseling could be an effective treatment for people with mental illnesses. Similar findings were reported from the studies done in Tanzania [39] and southern India [12]. In contrast, almost half of the participants perceived mental illness should be managed by witchdoctors and can be cured by marriage. Similar findings were reported from the studies done in India [12], Nigeria [33, 40], Ethiopia [3], and a study was done in developing countries [41]. These findings highlight the public misconceptions of mental problems and its treatment, as well as problems of accessibility and affordability of the services, might have contributed to the preference of treatment setting in this study. The need for dedicated and innovative efforts by mental health professionals to improve people’s awareness and attitudes towards mental health problems cannot be overemphasized.

In this study monthly income of the family was a predictor of attitude towards mental health problems. This is consistent with the findings from the European epidemiology of mental disorders [42], Saudi Arabia [26], and culture and mental health book [43]. This can show poverty and low-income can deprive people from accessing appropriate health care services.

In the current study, occupational status was associated with a favorable attitude towards mental health problems. Similar findings were reported from the survey done among Gimbi Town residents, Western Ethiopia [9], and the Saudi public [26]. Employed people may have more exposure to awareness on mental health problems which may improve their perceptions and attitudes towards mental illness.

In the current study, knowledge status was not a predictor of attitude towards mental health and problems. Contrary to this, studies done in Saudi Arabia, Lebanon, and Gimbi town Ethiopia, have shown a positive correlation of knowledge and attitudes with mental health problems [9, 20, 37]. However, this study analysis result has showed that 127(56.4%) had a favorable attitude among those who had adequate knowledge. Similarly, among those who had inadequate knowledge, 99(50.8%) had an unfavorable attitude. All said and done, attitudes are difficult to change and are subject to social influences. There has to be an internal motivation to change. Constant and consistent awareness of mental health and illness, accessibility, availability, and affordability of mental health services could improve people’s attitudes towards mental health and mental health problems.

## Conclusion

This study has showed that a significant percentage of the respondents had an unfavorable attitude towards mental health problems, and many of the respondents preferred religious and traditional treatments. This suggests the need to design effective community-based mental health interventions to improve the general public attitude and help-seeking behavior towards mental health and mental health problems. Additionally, the finding of this study may help clinicians working in the areas of mental health to have a better understanding of the community perception towards mental health problems and to design locally sound health education strategies to address the gaps in their day to day clinical practices. Lastly, further research needs to be conducted to highlight factors contributing to community attitude and help-seeking behavior in the study setting.

## Supporting information

S1 File Dataset. (SAV)
